# Three Years of Human Milk Banking: Assessing the Impact on Lactation Rates at Discharge in VLBW Preterm Infants in an Italian Reference NICU

**DOI:** 10.3390/nu17091440

**Published:** 2025-04-25

**Authors:** Federica Mongelli, Andrea Calandrino, Francesco Vinci, Cristina Traggiai, Daniela Rebora, Elena Maggiora, Luca Antonio Ramenghi

**Affiliations:** 1 Neonatal Intensive Care Unit, Department of Maternal and Neonatal Health, IRCCS Giannina Gaslini Institute, 16147 Genoa, Italy; federicamongelli@gaslini.org (F.M.); francescovinci@gaslini.org (F.V.); lucaramenghi@gaslini.org (L.A.R.); 2 Department of Neonatology, International Evangelic Hospital, 16158 Genoa, Italy; cristina.traggiai@ospedale-evangelico.it; 3 Donor Human MilkBank, Medical Directorate, IRCCS Giannina Gaslini Institute, 16147 Genoa, Italy; danielarebora@gaslini.org; 4 Neonatal Intensive Care Unit, Department of Public Health and Pediatrics, University of Turin, 10127 Turin, Italy; elena.maggiora@gmail.com; 5 A.O.U. Città della Salute e della Scienza di Torino, 10127 Turin, Italy; 6 Department of Neuroscience, Rehabilitation, Ophthalmology, Genetics, Mother and Child Health, School of Medical and Pharmaceuticals, University of Genoa, 16132 Genoa, Italy

**Keywords:** neonates, human milk bank, preterm infants, feeding practices, VLBW

## Abstract

**Background:** Human milk (HM) offers critical short- and long-term benefits for preterm and very low birth weight (VLBW) infants. In 2021, a human milk bank (HMB) was established at the IRCCS Giannina Gaslini Institute, aiming to improve HM feeding rates in this vulnerable population. **Methods:** We retrospectively analyzed feeding data from 442 VLBW infants (BW < 1500 g) admitted between 2018 and 2024. Data were drawn from the Vermont Oxford Network and Italian Neonatal Network registries. Feeding modalities—exclusive HM, infant formula milk (IM), and mixed feeding (MF)—were recorded and analyzed before and after HMB introduction. **Results:** Before 2021, MF was predominant, with exclusive HM rates below 10%. Following HMB implementation, exclusive HM feeding increased significantly, reaching 47.2% in 2024 (*p* < 0.0001). Regression analysis showed a positive trend for HM (+4.84%/year, *p* = 0.05), and a declining trend for IM (−1.96%/year) and MF (−2.88%/year). Projections suggest HM rates may exceed 58% by 2030. **Conclusions:** The introduction of the HMB was associated with a significant shift in feeding practices, increasing HM use and reducing IM exposure among VLBW infants. These findings underscore the importance of institutional strategies such as donor milk availability, lactation support, and maternal education in optimizing neonatal nutrition. Ongoing efforts are essential to sustain and extend these improvements beyond NICU discharge, ensuring the long-term benefits of human milk for preterm infants.

## 1. Introduction

Human milk is the optimal feeding choice for all newborns, including preterm and very low birth weight (VLBW) infants, due to its short- and long-term developmental and health benefits [1,2,3,4].

Human milk and particularly mother’s own milk, reduces mortality, feeding intolerance, nosocomial infections, necrotizing enterocolitis (NEC), retinopathy of prematurity (ROP), and bronchopulmonary dysplasia (BPD) [5,6,7]. Mothers’ milk contains bioactive molecules, including anti-infective and growth factors and human milk oligosaccharides, which protect preterm infants [8].

Additionally, human milk is better digested by the immature gastrointestinal system, enabling faster achievement of full enteral feeding than formula. This leads to reduced exposure to central vascular lines and their associated infectious risks [9,10]. A dose–response relationship has been observed, indicating that higher consumption of human milk correlates with reduced morbidity [11].

Long-term benefits include enhanced psychomotor development [12,13] and reduced cardiovascular risk factors [14].

On the other hand, formula feeding in preterm infants is associated with an increased risk of NEC. According to Lucas & Cole (1990) [15], NEC was 6–10 times more prevalent in exclusively formula-fed infants than in those receiving human milk. Every effort should be made to promote maternal lactation, but donor milk is often necessary for very preterm infants due to challenges in early maternal milk production [16]. Donated milk undergoes pasteurization, which alters its composition and affects the beneficial effects of human milk. However, it still represents the best alternative for premature infants when the mother’s own milk is not available.

Donor milk allows early enteral feeding initiation, avoiding the risks associated with formula [7,10,17,18,19,20].

Despite these benefits, few studies have investigated the use of donor milk in neonatal intensive care units (NICUs) and the impact of human milk banks on clinical practice and enteral feeding at discharge. A study in Tuscany, Italy, involving 6 donor milk banks and 25 hospitals, found that initiating enteral feeding with donor milk did not adversely affect the proportion of infants receiving mothers’ milk at full enteral feeding (FEF) [21]. However, the type of milk used during enteral feeding did not influence the feeding type at discharge, where 27% of infants were fed mothers’ milk—a proportion similar to that found in a prior study on Italian NICUs [22].

HMB initiatives have significantly improved neonatal nutrition by increasing access to HM, especially for VLBW and preterm infants. Moreover, introducing an HMB often promotes a shift in unit culture, enhancing the prioritization of HM use and encouraging staff and parental engagement through targeted education and support [21].

Newborns initially fed donor milk had the highest prevalence (91.3%) of exclusive human milk feeding at FEF.

Arslanoglu et al. (2013) [16] conducted a survey comparing breastfeeding rates at discharge among 83 Italian NICUs, 19 of which had milk banks and 64 that did not. Exclusive breastfeeding rates were significantly higher in NICUs with milk banks (29.6% vs. 16%).

In a multicenter study involving 12 level-3 NICUs and 594 VLBW infants, 30.5% were exclusively breastfed at discharge, 0.2% were predominantly breastfed, 23.8% received complementary feeding, and 45.5% were exclusively formula-fed. Exclusive breastfeeding rates varied widely among NICUs, ranging from 0% to 68.6% [22].

Similar trends have been reported in other countries, including Australia [23], the USA [24], and Spain [25], reinforcing the role of human milk banks in improving feeding practices in preterm infants.

This study aims to assess the impact of the opening of a human milk bank (HMB) in 2021 on human milk feeding rates at discharge for VLBW infants in an Italian level-3 NICU, a regional reference center, by collecting data over the following three years. We hypothesized that introducing an HMB would lead to an increase in exclusive HM feeding at discharge and a decline in infant formula use over time.

## 2. Materials and Methods

### 2.1. Patient Data and Feeding Records

We considered feeding modalities at discharge for VLBW infants (born with a birth weight [BW] <1500 g) who were admitted to the NICU of the IRCCS Giannina Gaslini Institute, a tertiary-level academic reference center for newborns. We excluded from our analysis patients who died during the period of in-hospital stay.

Data about BW, gestational age (GA), and feeding modalities were obtained from the Vermont Oxford Network (VON) (for the period 2018–2020) and Italian Neonatal Network of the Italian Society of Neonatology (INNSIN) (for the period 2021–2024) records. We registered the type of milk given at discharge. We categorized the VLBW infants as exclusive mother’s own milk (HM) receivers if only mother’s own milk was given, as infant formula only (IM) if no mother’s own milk was available, and mixed feeding (MF) if the infant was discharged with any amount of both human and formula milk.

### 2.2. Nutritional Management

Per our departmental protocol, parenteral nutrition is initiated immediately after establishing central access at birth. Minimal enteral feeding (MEF) begins within 24 h after birth, with initial volumes of 10 mL/kg/day for neonates weighing less than 1000 g and 20 mL/kg/day for those over 1000 g. After three days of MEF, the enteral milk volume is gradually increased by 10–20 mL/kg every 48 h until the target volume of 160–180 mL/kg/day is reached. Maternal milk is the preferred source for enteral feeding. In its absence, formula milk was used before opening of the human milk bank (HMB).

Following the opening of the HMB, donor milk has always been used to start enteral feeding for every VLBW infant, according to parental consent.

In case of limited donor milk supply, after the infant reaches a body weight >1500 g, infant formula replaces donor milk if maternal milk is unavailable. If neither donor nor maternal milk is available, minimal enteral feeding (MEF) is initiated using a type 1 formula (term infant formula), which is then replaced with a preterm formula once the enteral feeding is about 80–100 mL/kg. Our HMB has, however, been able to cope with donor milk requests for VLBW infants since its opening.

When the enteral intake reaches 80 mL/kg/day, maternal or donor milk is fortified as needed with a bovine-derived human milk fortifier (Aptamil BMF) containing extensively hydrolyzed proteins, LCPUFAs (including DHA and AA), and essential micronutrients. As enteral feeding increases, parenteral nutrition is gradually reduced, with parenteral lipids discontinued once an enteral intake of 90–100 mL/kg/day is achieved. In contrast, parenteral amino acids and the 10% glucose solution are maintained.

Moreover, in conjunction with the opening of the HMB, targeted training sessions were provided to medical and nursing staff. These sessions aimed to ensure proper handling, storage, and administration of donor human milk and to promote awareness of human milk’s clinical benefits. The training was conducted before and during the initial implementation phase of the HMB to support a smooth integration into clinical practice.

### 2.3. Statistical Analysis

Descriptive statistics were computed for each feeding category, including means, medians, standard deviations, quartiles, and percentage distributions. For the analyses, we considered a period of 7 years, dividing the infants into two subgroups, considering 2021 as the splitting point, when the HMB was opened.

A chi-square test for independence was conducted to assess whether the distribution of feeding types changed significantly over time, comparing feeding trends pre-2021 (2018–2020) and post-2021 (2021–2024).

To examine feeding trends, linear regression analysis was performed, estimating the annual rate of change (slope) for each feeding type (% per year) and evaluating the statistical significance (*p*-value) and strength of association (R^2^ value).

Future projections were generated for the years 2025 to 2030, assuming historical trends would continue linearly. The model was fit using the least squares method, and predicted values were computed based on extrapolated trends.

All the analyses are based on two-tailed tests; the significance threshold was set at 5%.

All statistical analyses were conducted using Python 3.11.8 (pandas, statsmodels, and scipy modules for analyses, and seaborn and matplotlib for visualization).

## 3. Results

Out of a total VLBW cohort of 499 newborns, 442 patients were considered for the period 2018–2024 (57 patients died during hospitalization and were not considered according to the exclusion criteria), with a mean BW of 1093 ± 315.7 g and a median GA of 29 weeks (27–31). Details on the cohort clinical characteristics are reported in Table 1, dividing the population into the pre- and post-HMB opening period (splitting point 2021).

All patients were fed following the protocol of our NICU. We report that in the period 2018–2020, IM was widely prescribed at discharge, showing fluctuations but remaining relatively stable, while HM remained low and inconsistent, averaging below 10%. MF was the dominant feeding practice during this period. However, a clear shift in feeding trends emerged after introducing the human milk bank (HMB) in 2021. IM began to decline, while HM significantly increased, reaching 47.2% in 2024, indicating a growing preference for exclusive human milk feeding. Although MF has remained the most common practice, it has declined recently. Details in feeding rates per year are reported in Table 2.

The chi-square test results, obtained by comparing different feeding modalities around the 2021 milestone, corroborate the result that feeding practices significantly changed after the introduction of the HMB (χ^2^ = 31.26, *p* < 0.0001), while no significant differences were found in the pre-2021 period (χ^2^ = 5.38, *p* = 0.25).

**Figure 1:**
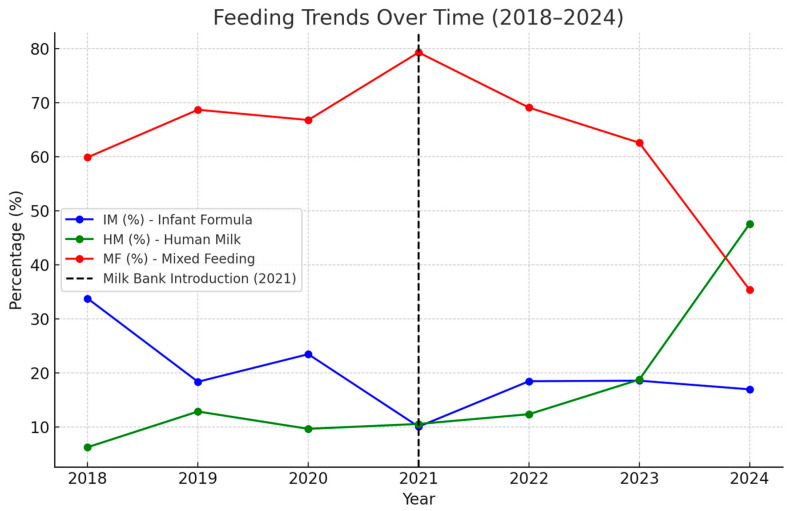
Feeding trends before and after HMB introduction (2018–2024). HMB, human milk bank; HM, human milk; IM, infant formula milk; MF, mixed feeding.

Regression analyses further confirmed a strong increasing trend in HM consumption (+4.84% per year, *p* = 0.05, R^2^ = 0.57), suggesting a significant positive impact of the HMB. Conversely, IM (−1.96% per year, *p* = 0.162, R^2^ = 0.35) and MF (−2.88% per year, *p* = 0.296, R^2^ = 0.21) exhibited declining trends, though they were not statistically significant (Figure 1).

In the end, no significant differences were found in the mean in-hospital stay duration when comparing the pre-2021 and post-2021 subcohorts (χ^2^ = 5.23, *p* = 0.32).

### Predictive Analysis

**Figure 2:**
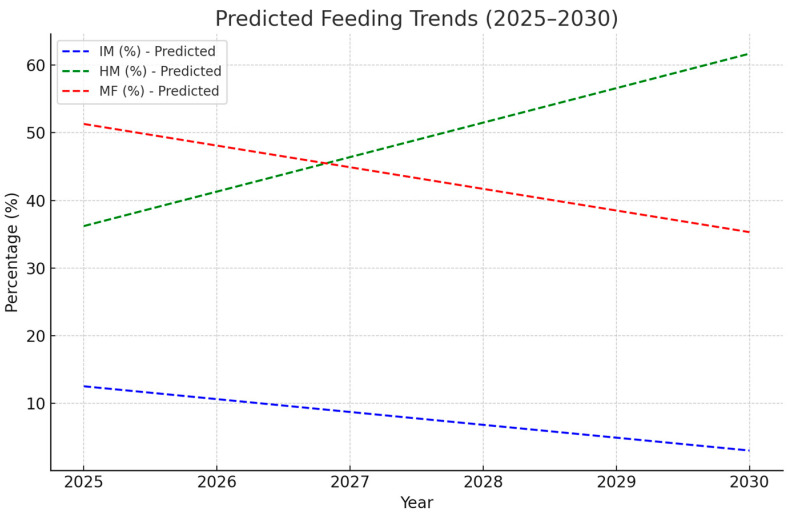
Predicted feeding trends for the period 2025−2030. HMB, human milk bank; HM, human milk; IM, infant formula milk; MF, mixed feeding.

The predictive analysis, conducted based on the existing data, estimates that by 2030, the proportion of infants receiving HM will reach approximately 58.4%, reflecting an annual increase of +4.8% annually. Conversely, the use of IM is expected to decline to 11.2%, following a negative trend of −1.96% per year. MF is projected to decrease further, dropping to 30.4% by 2030. Predicted trend lines are shown in Figure 2.

## 4. Discussion

The establishment of a human milk bank (HMB) in 2021 and subsequent intensive promotion efforts to encourage mothers to become milk donors have significantly modified feeding practices among VLBW preterm infants.

Our data confirm a substantial shift over the past seven years, particularly following the introduction of the HMB, with a marked decline in infant formula milk (IM), a substantial increase in human milk (HM), and a fluctuating yet gradually declining presence of mixed feeding (MF). Specifically, IM has decreased over time, with a mean of 20.12%, although showing variability across years, aligning with previous findings that exposure to formula has been significantly reduced with the implementation of donor milk policies [26,27].

In contrast, HM has shown a strong upward trend, reaching 47.17% in 2024, the highest recorded value, reflecting findings from Spatz et al. (2020) [28] and Wilunda et al. (2023) [29] that comprehensive lactation support can improve maternal milk provision during NICU hospitalization and beyond. This suggests that, in addition to the availability of donor human milk, the active promotion and education efforts following the HMB introduction, particularly in motivating mothers to feed their children and to donate their milk, may have played a crucial role in influencing maternal choices and increasing HM rates at discharge.

The increase in HM rates may also reflect an improved sensitivity to human milk benefits among our intensively retrained staff nurses and doctors.

While MF remains the dominant feeding method, with a mean of 63.12%, it exhibited a decline in 2024, suggesting a potential shift toward exclusive human milk feeding in line with strategies to promote exclusive breastfeeding at discharge [16].

The increasing proportion of HM-fed infants in 2024 further supports the evidence that institutional interventions, such as prenatal lactation consultations, individualized maternal support, and physician education, are essential for enhancing breastfeeding success [30].

We did not report any significant differences in the length of the hospitalization period comparing the pre- and post-HMB opening. Given the reduced sample size, we argue that too many clinical biases may have occurred, modifying the hospital period duration independently from the FEF reaching time.

Moreover, the predictive analysis suggests that if current trends persist, HM consumption will continue to rise, potentially exceeding 58% by 2030, while IM and MF usage will decline. In its current form, the predictive model is limited by the assumption of a linear positive correlation based on retrospective data collected over a relatively short time span. This approach does not account for potential external influences, such as policy changes, public health initiatives, or shifts in clinical practice, that could impact feeding trends in the future. Given these limitations, our findings support the idea that ongoing education and targeted recruitment efforts to increase milk donor participation may be instrumental in reinforcing the benefits of human milk feeding and sustaining this trajectory.

Despite these improvements, challenges remain in maintaining long-term breastfeeding success post-discharge. While our data indicate a clear trend favoring human milk, further efforts should aim to increase HM rates at discharge, which are below the desirable rate, and increase rates beyond the NICU stay through structured outpatient follow-ups and community lactation support. As demonstrated in previous research, a multidisciplinary approach integrating lactation counseling, maternal education, and healthcare provider training is essential for sustaining exclusive human milk feeding [31,32].

Our findings reinforce the role of HMBs as a key intervention in shaping feeding practices, ensuring that preterm infants receive optimal nutrition while reducing exposure to artificial formula and its associated risks [33,34]. As predictive trends suggest a continued rise in HM feeding, further structured support programs and sustained efforts to encourage milk donation will ensure these improvements are maintained over time.

The shift in feeding trends after introducing the HMB can be better understood using models of implementation science and behavior change. In particular, the COM-B model (Capability, Opportunity, Motivation—Behavior) helps explain how staff training, more access to donor human milk, and institutional support increased healthcare providers’ ability and motivation to prioritize its use. These models suggest that the HMB not only served as a structural intervention but also triggered cultural and behavioral changes in the neonatal unit [35].

This study has several limitations. First, we could not analyze maternal breastfeeding rates before 2021, as this variable was not recorded in the VON database before that time. Second, the study’s retrospective nature limits the ability to establish causal relationships. Additionally, the sample size was relatively small, and the period following the opening of the human milk bank was limited to only a few years, which may not fully capture long-term trends and outcomes.

## 5. Conclusions

Our study provides compelling evidence that introducing an HMB, combined with comprehensive promotion and education efforts, has significantly altered feeding practices in NICUs and milk supply at discharge. Continued commitment to enhancing breastfeeding support, expanding donor participation, and strengthening lactation education will ensure that human milk remains the standard of care for preterm infants, ultimately improving health outcomes for this vulnerable population.

## Figures and Tables

**Table 1:** Population clinical characteristics.

Subcohort	Total Patients	GA (Median, 25–75°)	*p*-Value	BW (Mean ± SD)	*p*-Value
Pre-HMB *	208	28.5 (26.5–30)	0.18	1065 g ± 325 g	0.27
Post-HMB *	234	29.5 (28–31.5)		1112 g ± 308 g	

HMB, human milk bank; *, HMB opening year 2021; GA, gestational age; BW, birth weight.

**Table 2:** Patient number and feeding rates per year.

Year	Total Patients	HM (%)	IM (%)	MF (%)
2018	74	5 (6.8)	25 (33.8)	44 (59.4)
2019	71	9 (12.7)	13 (18.3)	49 (69)
2020	63	6 (9.5)	15 (23.8)	42 (66.7)
2021	47	5 (10.6)	5 (10.6)	37 (78.8)
2022	59	7 (11.8)	11 (16.6)	41 (71.6)
2023	75	14 (18.6)	14 (18.6)	47 (62.8)
2024	53	25 (47.2)	9 (17)	19 (35.8)

HM, human milk; IM, infant formula; MF, mixed feeding.

## Data Availability

The data presented in this study are available upon request from the corresponding author due to privacy and ethical restrictions.

## Abbreviations

The following abbreviations are used in this manuscript:

HM	Human milk
IM	Infant formula milk
MF	Mixed feeding
HMB	Human milk bank
VLBW	Very low birth weight
NICU	Neonatal intensive care unit
NEC	Necrotizing enterocolitis
ROP	Retinopathy of prematurity
BPD	Bronchopulmonary dysplasia
BW	Birth weight
GA	Gestational age
VON	Vermont Oxford Network
INNSIN	Italian Neonatal Network of the Italian Society of Neonatology
MEF	Minimal enteral feeding
LCPUFAs	Long-chain polyunsaturated fatty acids
DHA	Docosahexaenoic acid
AA	Arachidonic acid
FEF	Full enteral feeding

## References

[1] Breastfeeding S.O., Eidelman A.I., Schanler R.J., Johnston M., Landers S., Noble L. (2012). Breastfeeding and the Use of Human Milk. Pediatrics.

[2] Bernardo H., Cesar V. (2013). Long-Term Effects of Breastfeeding: A Systematic Review.

[3] Underwood M.A. (2013). Human milk for the premature infant. Pediatr. Clin. N. Am..

[4] Moro G.E., Arslanoglu S., Bertino E., Corvaglia L., Montirosso R., Picaud J., Polberger S., Schanler R.J., Steel C., van Goudoever J. (2015). XII. Human Milk in Feeding Premature Infants: Consensus Statement. J. Pediatr. Gastroenterol. Nutr..

[5] Flidel-Rimon O., Friedman S., Lev E., Juster-Reicher A., Amitay M., Shinwell E.S. (2004). Early enteral feeding and nosocomial sepsis in very low birthweight infants. Arch. Dis. Child. Fetal Neonatal Ed..

[6] Meier P.P., Engstrom J.L., Patel A.L., Jegier B.J., Bruns N.E. (2010). Improving the use of human milk during and after the NICU stay. Clin. Perinatol..

[7] Sisk P.M., Lovelady C.A., Dillard R.G., Gruber K.J., O’Shea T.M. (2007). Early human milk feeding is associated with a lower risk of necrotizing enterocolitis in very low birth weight infants. J. Perinatol..

[8] Bode L. (2018). Human milk oligosaccharides in the prevention of necrotizing enterocolitis: A journey from in vitro and in vivo models to mother-infant cohort studies. Front Pediatr.

[9] Corpeleijn W.E., De Waard M., Christmann V., Van Goudoever J.B., Jansen-Van Der Weide M.C., Kooi E.M.W., Koper J.F., Kouwenhoven S.M.P., Lafeber H.N., Mank E. (2016). Effect of Donor Milk on Severe Infections and Mortality in Very Low-Birth-Weight Infants: The Early Nutrition Study Randomized Clinical Trial. JAMA Pediatr..

[10] Schanler R.J., Lau C., Hurst N.M., Smith E.O.B. (2005). Randomized trial of donor human milk versus preterm formula as substitutes for mothers’ own milk in the feeding of extremely premature infants. Pediatrics.

[11] Miller J., Tonkin E., Damarell R.A., McPhee A.J., Suganuma M., Suganuma H., Middleton P.F., Makrides M., Collins C.T. (2018). A Systematic Review and Meta-Analysis of Human Milk Feeding and Morbidity in Very Low Birth Weight Infants. Nutrients.

[12] Blesa M., Sullivan G., Anblagan D., Telford E.J., Quigley A.J., Sparrow S.A., Serag A., Semple S.I., Bastin M.E., Boardman J.P. (2019). Early breast milk exposure modifies brain connectivity in preterm infants. Neuroimage.

[13] Lucas A., Morley R., Cole T.J., Gore S.M. (1994). A randomised multicentre study of human milk versus formula and later development in preterm infants. Arch. Dis. Child. Fetal Neonatal Ed..

[14] Singhal A., Cole T.J., Lucas A. (2001). Early nutrition in preterm infants and later blood pressure: Two cohorts after randomised trials. Lancet.

[15] Lucas A., Cole T.J. (1990). Breast milk and neonatal necrotising enterocolitis. Lancet.

[16] Arslanoglu S., Corpeleijn W., Moro G., Braegger C., Campoy C., Colomb V., Decsi T., Domellöf M., Fewtrell M., ESPGHAN Committee on Nutrition (2013). Donor human milk for preterm infants: Current evidence and research directions. J. Pediatr. Gastroenterol. Nutr..

[17] Schanler R.J., Shulman R.J., Lau C., Smith E.O.B., Heitkemper M.M. (1999). Feeding strategies for premature infants: Randomized trial of gastrointestinal priming and tube-feeding method. Pediatrics.

[18] Menon G., Williams T.C. (2013). Human milk for preterm infants: Why, what, when and how?. Arch. Dis. Child. Fetal Neonatal Ed..

[19] Meinzen-Derr J., Poindexter B., Wrage L., Morrow A.L., Stoll B., Donovan E.F. (2009). Role of human milk in extremely low birth weight infants’ risk of necrotizing enterocolitis or death. J. Perinatol..

[20] Sullivan S., Schanler R.J., Kim J.H., Patel A.L., Trawöger R., Kiechl-Kohlendorfer U., Chan G.M., Blanco C.L., Abrams S., Cotten C.M. (2010). An exclusively human milk-based diet is associated with a lower rate of necrotizing enterocolitis than a diet of human milk and bovine milk-based products. J. Pediatr..

[21] Berti E., Puglia M., Perugi S., Gagliardi L., Bosi C., Ingargiola A., Magi L., Martelli E., Pratesi S., Sigali E. (2018). Feeding Practices in Very Preterm and Very Low Birth Weight Infants in an Area Where a Network of Human Milk Banks Is in Place. Front. Pediatr..

[22] Davanzo R., Monasta L., Ronfani L., Brovedani P., Demarini S. (2013). Breastfeeding at NICU discharge: A multicenter Italian study. J. Hum. Lact..

[23] Simmer K., Hartmann B. (2009). The knowns and unknowns of human milk banking. Early Hum. Dev..

[24] Montgomery D., Schmutz N., Baer V.L., Rogerson R., Wheeler R., Rowley A.-M., Lambert D.K., Christensen R.D. (2008). Effects of Instituting the “BEST Program” (Breast Milk Early Saves Trouble) in a Level III NICU. J. Hum. Lact..

[25] Utrera Torres M.I., Medina López C., Vázquez Román S., Díaz C.A., Cruz-Rojo J., Cooke E.F., Alonso C.R.P. (2010). Does opening a milk bank in a neonatal unit change infant feeding practices? A before and after study. Int. Breastfeed. J..

[26] Marinelli K.A. (2020). Wet Nurses to Donor Milk Banks and Back Again: The Continuum of Sharing Our Milk to Save Lives. J. Hum. Lact..

[27] Vázquez-Román S., Bustos-Lozano G., López-Maestro M., Rodríguez-López J., Orbea-Gallardo C., Samaniego-Fernández M., Pallás-Alonso C. (2014). Clinical impact of opening a human milk bank in a neonatal unit. An. Pediatría (Engl. Ed.).

[28] Spatz D.L. (2020). Using the Coronavirus Pandemic as an Opportunity to Address the Use of Human Milk and Breastfeeding as Lifesaving Medical Interventions. JOGNN-J. Obstet. Gynecol. Neonatal Nurs..

[29] Wilunda C., Israel-Ballard K., Wanjohi M., Lang’At N., Mansen K., Waiyego M., Kibore M., Kamande E., Zerfu T., Kithua A. (2024). Potential effectiveness of integrating human milk banking and lactation support on neonatal outcomes at Pumwani Maternity Hospital, Kenya. Matern. Child Nutr..

[30] Murphy L., Warner D.D., Parks J., Whitt J., Peter-Wohl S. (2014). A quality improvement project to improve the rate of early breast milk expression in mothers of preterm infants. J. Hum. Lact..

[31] Bertino E., Giuliani F., Baricco M., Di Nicola P., Peila C., Vassia C., Chiale F., Pirra A., Cresi F., Martano C. (2013). Benefits of donor milk in the feeding of preterm infants. Early Hum. Dev..

[32] Adhisivam B., Kohat D., Tanigasalam V., Bhat V., Plakkal N., Palanivel C. (2019). Does fortification of pasteurized donor human milk increase the incidence of necrotizing enterocolitis among preterm neonates? A randomized controlled trial. J. Matern. Fetal Neonatal Med..

[33] Parker M.G.K., Burnham L., Mao W., Philipp B.L., Merewood A. (2016). Implementation of a Donor Milk Program Is Associated with Greater Consumption of Mothers’ Own Milk among VLBW Infants in a US, Level 3 NICU. J. Hum. Lact..

[34] Pescador-Chamorro M.I., Caballero-Martín S., Rodríguez-Corrales E., Vigil-Vázquez S., Sánchez-Luna M. (2025). The Positive Effect on Preterm Infants’ Feeding of Human Milk During Hospitalization and at Discharge after the Opening of a Personalized Nutrition Unit. Breastfeed Med..

[35] Michie S., van Stralen M.M., West R. (2011). The behaviour change wheel: A new method for characterising and designing behaviour change interventions. Implement. Sci..

